# Immunomodulatory Properties of BRAF and MEK Inhibitors Used for Melanoma Therapy—Paradoxical ERK Activation and Beyond

**DOI:** 10.3390/ijms22189890

**Published:** 2021-09-13

**Authors:** Thomas Jung, Maximilian Haist, Michael Kuske, Stephan Grabbe, Matthias Bros

**Affiliations:** Medical Center, Department of Dermatology, University Mainz, Langenbeckstraße 1, 55131 Mainz, Germany; thjung@students.uni-mainz (T.J.); mhaist@uni-mainz.de (M.H.); mikuske@uni-mainz.de (M.K.); stephan.grabbe@unimedizin-mainz.de (S.G.)

**Keywords:** BRAF, MEK, targeted therapy, immunological effects, immune checkpoint inhibitors, metastatic melanoma, CTLA-4, PD-1, PD-L1, paradoxical ERK activation, inflammasome, tumor microenvironment

## Abstract

The advent of mitogen-activated protein kinase (MAPK) inhibitors that directly inhibit tumor growth and of immune checkpoint inhibitors (ICI) that boost effector T cell responses have strongly improved the treatment of metastatic melanoma. In about half of all melanoma patients, tumor growth is driven by gain-of-function mutations of BRAF (v-rat fibrosarcoma (Raf) murine sarcoma viral oncogene homolog B), which results in constitutive ERK activation. Patients with a BRAF mutation are regularly treated with a combination of BRAF and MEK (MAPK/ERK kinase) inhibitors. Next to the antiproliferative effects of BRAF/MEKi, accumulating preclinical evidence suggests that BRAF/MEKi exert immunomodulatory functions such as paradoxical ERK activation as well as additional effects in non-tumor cells. In this review, we present the current knowledge on the immunomodulatory functions of BRAF/MEKi as well as the non-intended effects of ICI and discuss the potential synergistic effects of ICI and MAPK inhibitors in melanoma treatment.

## 1. Introduction

Malignant melanoma is one of the most aggressive solid tumors of the skin and occurs at increasing incidence in the western world [1]. Despite major efforts in the prevention of melanoma, including skin cancer screening, up to a few years ago melanoma patients with distant metastases had poor prognoses with median survival rates of only six to ten months [2]. Over the past decade, significant progress has been made in the treatment of advanced malignant melanoma [3]. In particular, the approval of immune checkpoint inhibitors (ICI) that activate the patient’s immune system by promoting T effector cell generation and activation has significantly improved the prognosis of affected patients and demonstrated long-term efficacy in up to 50% of ICI treated patients [4,5].

The advent of mitogen-activated protein kinase (MAPK)-targeted therapy was another pivotal area in the improvement in melanoma treatment. In about 40–50% of patients, melanoma cells are characterized by a mutation of BRAF (v-rat fibrosarcoma (Raf) murine sarcoma viral oncogene homolog B) at position 600 (BRAF^V600^), which results in constitutive activation of the extracellular signal-regulated kinase (ERK) pathway [6]. In turn, hyperactive ERK promotes the proliferation and migration of tumor cells [7]. Therefore, BRAF inhibitors (BRAFi), which specifically inhibit mutated BRAF^V600E^, have been developed [8]. BRAFi inhibit ERK signal transduction and, thus, induce apoptosis of tumor cells [9]. Thus far, the BRAFi vemurafenib, dabrafenib, and encorafenib have been approved for treatment [10]. Due to the development of tumor resistance to the action of BRAFi monotherapy by various mechanisms and the risk of developing secondary cancers [11,12], combination therapies with MEK (MAPK/ERK kinase) inhibitors (MEKi) have been introduced to delay or prevent the onset of resistance [13].

A deeper understanding of the effects of inhibitors of the ERK pathway on the biology of malignant melanoma as well as the host immune system may provide new opportunities to improve the treatment of the tumor. For example, histopathologic examination of tissue biopsies from melanoma patients treated with BRAFi demonstrated increased densities of tumor-infiltrating leukocytes (TIL) [14]. In addition, T cell-stimulating effects by BRAFi were observed in vitro and in vivo, which might be attributed to paradoxical activation of the ERK pathway as described in more detail in Section 4.3. Above, we showed that murine bone marrow-derived dendritic cells (DC) responded to BRAFi application with an increased expression of T cell costimulatory receptors and elevated production of the pro-inflammatory cytokine IL-1β [15], which is suspected to promote tumor growth [16,17]. Similar findings were obtained for human monocyte-derived DC. Taken together, these results indicate that BRAFi exhibit relevant off-target effects in immune cells, which may play a considerable role in the context of therapy efficacy. This review aims to give insights into the immunological (off-target) effects of MAPK-targeted therapy, which in turn may impact the efficacy of treatment.

## 2. Constitutive MAPK Signaling Is a Major Cause of Melanoma Induction/Progression

### 2.1. BRAF Mutations Are a Dominant Driver of Melanoma

In about half of all melanoma patients gain-of-function mutations of the *BRAF* proto-oncogene [12] are apparent, followed by loss-of-function mutations of the *neurofibroma 1* (*NF1*) [18], and by gain-of-function mutations of the *neuroblastoma rat sarcoma (RAS) viral oncogene homolog* (*NRAS*) and the *c-KIT* proto-oncogene [19].

In recent years, it has further been found that the development of melanoma from preneoplastic lesions is not based on a specific mutation pattern. Rather, different gene mutations can transform different precursor lesions into each melanoma subtype [20]. Therefore, a BRAF mutation alone, as frequently found in melanocytic nevi, is not sufficient for melanoma development but might rather be complemented by additional mutations [21]. Here, UV radiation and other factors, such as oxidative stress, can cause additional mutations of, for example, the promoter of *telomerase reverse transcriptase* (*TERT*), [22] which may result in an elevated TERT expression, or of the *cyclin-dependent kinase inhibitor 2A* (*CDKN2A*) gene [23], thus initiating a malignant transformation.

### 2.2. Dysregulated Signaling Pathways

In most cases of melanoma, the ERK pathway is constitutively activated and promotes melanoma development due to dysregulation of the cell cycle and apoptosis [24]. In about 60% of cutaneous melanomas caused by intermittent sun exposure, a genetic gain-of-function mutation of the BRAF gene causes hyperactivity of the downstream ERK kinase [25]. More than 97% of BRAF mutations are located in codon 600 of the *BRAF* gene. In up to 90%, a transversion of thymine to adenine occurs, which leads to the substitution of valine (V) by glutamic acid (E), yielding BRAF^V600E^. Substitutions of valine by lysine (V600K) occur in approximately 8–20% of cases [26]. The hydrophilic amino acid, glutamic acid, present in BRAF^V600E^ instead of the hydrophobic amino acid, valine, causes constitutive activation of the catalytic domain of BRAF serine/threonine protein kinase, resulting in a 500-fold increase in kinase activity compared with wild-type BRAF kinase [27]. Hyperactive MAPK signaling is also caused by mutations of NRAS (about 20% of malignant melanoma) [28]. In over 80% of cases, NRAS displays a point mutation at position 61, leading to decreased guanosine 5’-triphosphate (GTPase) activity and, thus, maintenance of the GTP-bound conformation of the RAS protein. As a result, both MAPK and PI3K (phosphoinositide 3-kinases) signaling maintains active [19]. Typically, either BRAF or NRAS mutations are found in malignant melanomas. These mutations are mutually exclusive at the level of individual cells but not at the tumor level [29].

The third most common mutated gene in malignant melanomas is the tumor suppressor gene *NF1* [18]. Mutations of *NF1* are clustered in patients exposed to chronic solar radiation and in elderly patients [30]. NF1 inhibits RAS signaling by regulating the conversion of active RAS-GTP to inactive RAS-GDP [31]. Accordingly, loss-of-function mutations of the NF1 protein result in hyper-activation of RAS and, in combination with mutant BRAF protein, prevent oncogene-induced senescence during melanoma development by deregulating the PI3K and MAPK pathways [32].

### 2.3. Additional Melanoma-Promoting Mutations

In addition to the aforementioned driver mutations, further mutations are found primarily at later stages of melanoma. These often affect TERT, CDKN2A, PTEN, and other proteins [33]. PTEN constitutes a tumor suppressor which plays a role in cell cycle regulation and inhibits cell invasion by antagonizing PI3K signaling [34]. In melanoma cell lines, a PTEN mutation rate of 30–40% has been observed, whereas in primary melanomas the frequency was about 10% [35]. In a considerable fraction of human melanomas, a combination of BRAF and PTEN mutations has been detected [36] resulting in parallel hyper-activation of the MAPK and PI3K pathways [37].

## 3. Melanoma-Induced Immune Modulation

**Figure 1:**
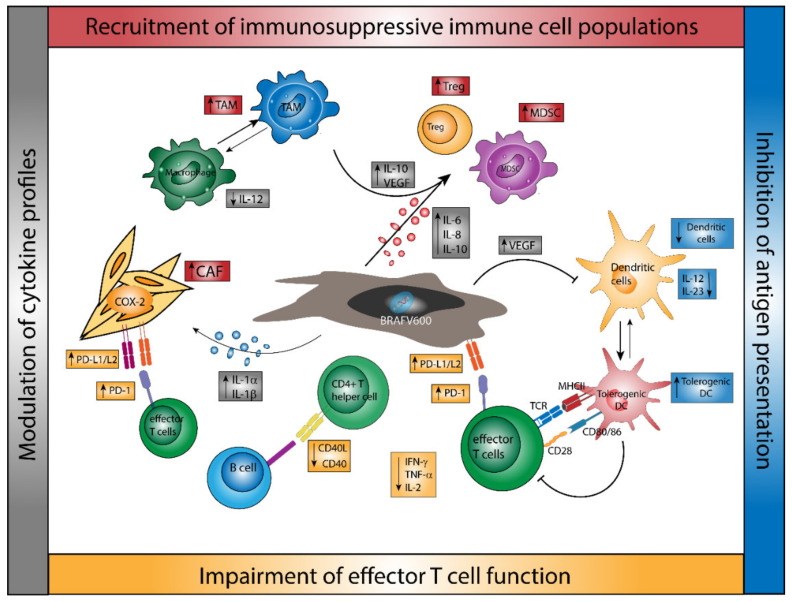
Immunological modulations of the tumor microenvironment in BRAF^V600^-mutated melanoma. BRAF^V600^-mutated melanoma is characterized by constitutive ERK activation, which results in an elevated release of immunomodulatory mediators that in turn affect both constituents of the microenvironment (TAM, CAF) and infiltrating immune cells (DC, T effector cells) to convey tumor immune evasion. BRAF-mutant melanoma is characterized by a modulation of cytokine profiles, which includes the accumulation of immunosuppressive cytokines such as IL-10 and VEGF, IL-1β, and lower levels of cytotoxic cytokines such as TNF-α and IFN-γ. Further, BRAFV600-mutated melanoma promote the recruitment of immunosuppressive cell populations such as regulatory T cells and myeloid-derived suppressor cells, favors macrophage polarization towards an M2-phenotype, which may inhibit anti-tumor immunity, and impedes the function of antigen-presenting cells. Accordingly, BRAF-mutant melanoma shows a strong impairment of effector T cell functions, which is conveyed by cell-cell contact-dependent mechanisms of immunosuppressive cell populations, the upregulation of PD-L1 on melanoma cells and cancer-associated fibroblasts, and a cytokine profile favoring the exhaustion and inhibition of effector T cells within the TME.

The development of melanomas is a multistep process that involves the interaction of environmental, genetic, and host factors. As such, melanoma-induced modulations of the tumor microenvironment (TME) contribute to immune evasion and melanoma progression. Intriguingly, BRAF-mutated melanomas may induce distinct alterations of the TME, which may serve as an additional mechanism of immune resistance (Figure 1). 

Bradley and co-workers have demonstrated that BRAF-mutated tumors downregulated HLA (major histocompatibility complex) class I molecules through its internalization and intracellular sequestration [38]. Furthermore, BRAF-mutated melanomas may create a TME that inhibits T cell effector functions even more effectively. In particular, it has been shown that developing tumors induced the accumulation of regulatory T cells (Treg) both in murine models of BRAF^V600^-mutant melanoma [39,40] and in human samples of BRAF^V600^-mutated melanoma [41], which may limit effector T cell activity via cell contact-dependent mechanisms or cytokine generation [42]. Moreover, Ho and co-workers reported that developing tumors in a BRAF^V600E^/PTEN^−/−^ murine melanoma model induced the recruitment and infiltration of myeloid-derived suppressor cells (MDSC), which are defined by their ability to suppress effector T cell functions [40]. This finding is consistent with previous observations of BRAF^V600^-mutant melanoma cells, which revealed a strong expression of IL-6 and IL-10, immunomodulatory cytokines that may promote the recruitment of MDSC and Treg to the TME [43]. 

Next, it has also been found that BRAF^V600^-mutant melanoma may prevent tumor antigen presentation by antigen-presenting cells (APC), such as macrophages and DC. Ott and co-workers demonstrated that cytokine generation and expression of activation markers in DC cocultured with BRAF-mutated melanoma cells were strongly impaired, while MAPK inhibition reversed this effect [44]. In agreement, Ho and co-workers showed that DC isolated from advanced BRAF-mutated melanomas were unable to stimulate the proliferation of CD8^+^ T cells [40]. Furthermore, during melanoma progression, the expression of CD40L on tumor-infiltrating CD4^+^ T cells, as well as the expression of interferon (IFN-)γ, tumor necrosis factor (TNF-)α, and IL-2 by these cells, was inhibited, accompanied by increased numbers of Treg and MDSC in the tumor. Notably, BRAF-mutated tumors did not only show an enhanced infiltration by immunosuppressive immune cell populations but Kim et al. have further observed the overexpression of genes associated with immunosuppression, such as cytotoxic T-lymphocyte associated protein (CTLA)-4, programmed cell death ligand (PD-L)1, or HLA-G [45].

In addition, it has been demonstrated that BRAF^V600E^ melanoma cells show an enhanced expression of IL-1α and IL-1β, which promoted the immunosuppressive properties of cancer-associated fibroblasts (CAF) [46]. Specifically, treatment of CAF isolated from melanoma patients with IL-1α/β strongly inhibited the proliferation and function of melanoma-specific cytotoxic T cells, which was attributed to an upregulation of cyclooxygenase-2 and increased surface expression of PD-L1 and PD-L2. Moreover, it has been found that BRAF^V600E^-mutant human melanoma cell lines increased the expression of vascular endothelial growth factor (VEGF), which may favor a tolerogenic DC phenotype and tumor progression by neoangiogenesis [43,47]. Inhibition of BRAF/MEK signaling by silencer (si)RNA and pharmacological inhibition decreased the production of pro-tumorigenic factors such as IL-1α/β [46], IL-6 [43], IL-8 [48], IL-10 [43,49], and VEGF [43,50] by melanoma cells. The transduction of melanoma cells with plasmids encoding BRAF^V600E^-specific short hairpin RNA significantly reduced the inhibitory properties of melanoma cell culture supernatants on the production of pro-inflammatory cytokines such as IL-12 and TNF-α by lipopolysaccharide (LPS)-stimulated DC [43]. Overall, these studies show that the constitutive ERK activation, as found in BRAF-mutated melanomas, may favor an ineffective anti-tumor immune response, indicating that a targeted inhibition of MAPK signaling may reverse these immunosuppressive effects [42] as outline below. 

## 4. MAPK-Targeted Treatment of Metastatic Melanoma

The majority of patients with newly diagnosed malignant melanoma are in the early stages of disease and are treated by excision of the lesion with an appropriate safety margin, based on the tumor thickness and the potential involvement of a sentinel lymph node [51]. After primary treatment, approximately 20% of patients relapse associated with metastases, for which the median survival without treatment would be less than 12 months. Until 2011, no systemic therapy was available that could convincingly improve survival in patients with metastatic melanoma [52]. With the approval of MAPK-targeted therapies, as well as the introduction of ICI, the median overall survival of these patients has been increased to more than 3 years [53].

### 4.1. Limitations of BRAFi Monotherapy

Prior to the approval of BRAFi for treatment, patients with BRAF^V600^-mutated melanoma had a worse prognosis than melanoma patients with wild-type BRAF [54]. The approval of MAPK-targeting inhibitors for the treatment of metastatic BRAF^V600E^-mutated melanoma changed this situation [55]. However, when BRAFi were administered alone, serious adverse events (AE) such as squamous cell carcinoma or keratoacanthoma occurred due to paradoxical activation of the ERK pathway [56]. In this regard, it is important to note that the currently clinically applied BRAFi may also cause homodimerization of wild-type BRAF or heterodimerization of BRAF with RAF1, resulting in unwanted BRAF activation in these cells, termed paradoxical ERK activation (see Section 4.3).

Moreover, BRAFi monotherapy almost invariably resulted in tumor resistance by various mechanisms [57]. For example, melanomas with a BRAF^V600E^ mutation may escape BRAF inhibition through RAS-mediated reactivation of the MAPK pathway, such as mutational inactivation of the tumor suppressor NF1 [58]. Another explanation for the development of BRAFi resistance is that due to the disrupted negative feedback mechanism associated with BRAF blockade, receptor tyrosine kinase (RTK) and RAS are activated, which in turn trigger the MAPK pathway [59]. In addition, RAF inhibitor-resistant RAF dimers form in this process, leading to the restoration of ERK activity. Moreover, the increased formation of BRAF dimers by RAS-independent alternative splicing [60] and mutations of MEK [61] have been demonstrated as additional mechanisms of resistance. Finally, tumors were reported to circumvent BRAF inhibition by allowing MEK signaling to restore activation of ERK in a BRAF-independent manner [62].

Besides the resistance to BRAFi autonomously acquired by tumor cells, the existing TME may also contribute to the development of BRAFi resistance [63]. For example, stromal cell secretion of hepatocyte growth factor (HGF) and subsequent activation of the HGF tyrosine kinase receptor can reactivate the MAPK and PI3K pathways [64]. Furthermore, BRAFi-induced paradoxical ERK activation in CAF was shown to result in elevated matrix protein production and remodeling [65]. The increased stiffness of the extracellular matrix (ECM) enhanced integrin-β1/focal adhesion kinase/Sarcoma signaling in melanoma cells, which in turn yielded ERK reactivation in a BRAF-independent manner. Additionally, the interplay of TAM, CAF, and tumor cells, based on the secretion of IL-1β by TAM, may mediate tolerance to BRAFi as well as to combination therapy with BRAFi and MEKi [66]. Paradoxical activation of the ERK pathway by BRAFi (as described in more detail in Section 4.3) in TAM was also shown to contribute to increased production of VEGF, which in turn reactivated the ERK pathway in melanoma cells, supporting tumor cell growth [67].

As a consequence of the several limitations of BRAFi, but especially the frequently apparent emergence of tumor resistance and associated tumor progression in patients, MEKi were developed and are co-applied in clinical practice to circumvent these problems [68].

### 4.2. BRAFi/MEKi Combination Therapy

In several clinical trials, combined treatment of patients with BRAFi and a MEKi (vemurafenib + cobimetinib [69]; dabrafenib + trametinib [70]; encorafenib + binimetinib [71]) increased progression-free survival (PFS) as compared with the according BRAFi monotherapy. However, despite the introduction of BRAFi/MEKi combination therapy, tumor resistance due to enhanced or combined evasion mechanisms continues to be observed. For example, *Braf* gene ultra-amplification leads to dimerization of BRAF with RAF1 and interaction of mutant MEK proteins with overexpressed BRAF, which consequently initiates reactivation of the MAPK pathway [72]. Additionally, for melanoma cells with double BRAFi/MEKi resistance, p21-activated kinases (PAK) were reported to become activated and to stimulate mechanistic targets of rapamycin signaling via c-Jun N-terminal protein kinase and β-catenin, inhibiting apoptosis in an ERK-independent manner [73]. Nevertheless, BRAFi/MEKi combination therapies are superior to BRAFi monotherapy and are, therefore, used as standard therapy in the treatment of BRAF^V600E^ metastatic malignant melanoma [74].

Currently, the evaluation of new inhibitors of the RAF/MEK/ERK signaling axis is the subject of preclinical research and is being investigated in clinical trials [75]. While dabrafenib, vemurafenib, and encorafenib belong to the second-generation of BRAFi and are all αC-OUT-RAF inhibitors (leading to a decrease in inhibitor binding to the other protomer of the dimer), the group of third generation BRAFi consists of both αC-OUT-RAF inhibitors [76] and αC-IN-RAF inhibitors (not strongly affecting inhibitor binding to the other monomeric subunit of the dimer) [77]. In addition to the biochemical differences present, third generation αC-OUT-RAF inhibitors are expected to retain the therapeutic breadth of second-generation αC-OUT-RAF inhibitors without promoting the appearance of secondary tumors through paradoxical ERK activation in healthy cells. However, the efficacy of αC-IN-RAF inhibitors is expected to be limited by a narrow therapeutic window because they inhibit ERK signaling in healthy cells as well. To overcome this phenomenon and ERK reactivation by feedback mechanisms, the combination of αC-IN-RAF inhibitors with αC-OUT-RAF inhibitors is expected to provide a remedy and ensure sustained inhibition of ERK [78].

Other side effects, such as rash and increased photosensitivity with vemurafenib, and pyrexia, hyperkeratosis, and headache with dabrafenib, are likely due to drug-specific off-target effects [79] as outlined in the following.

### 4.3. Off-Target Effects of BRAFi/MEKi on Immune Cells

**Figure 2:**
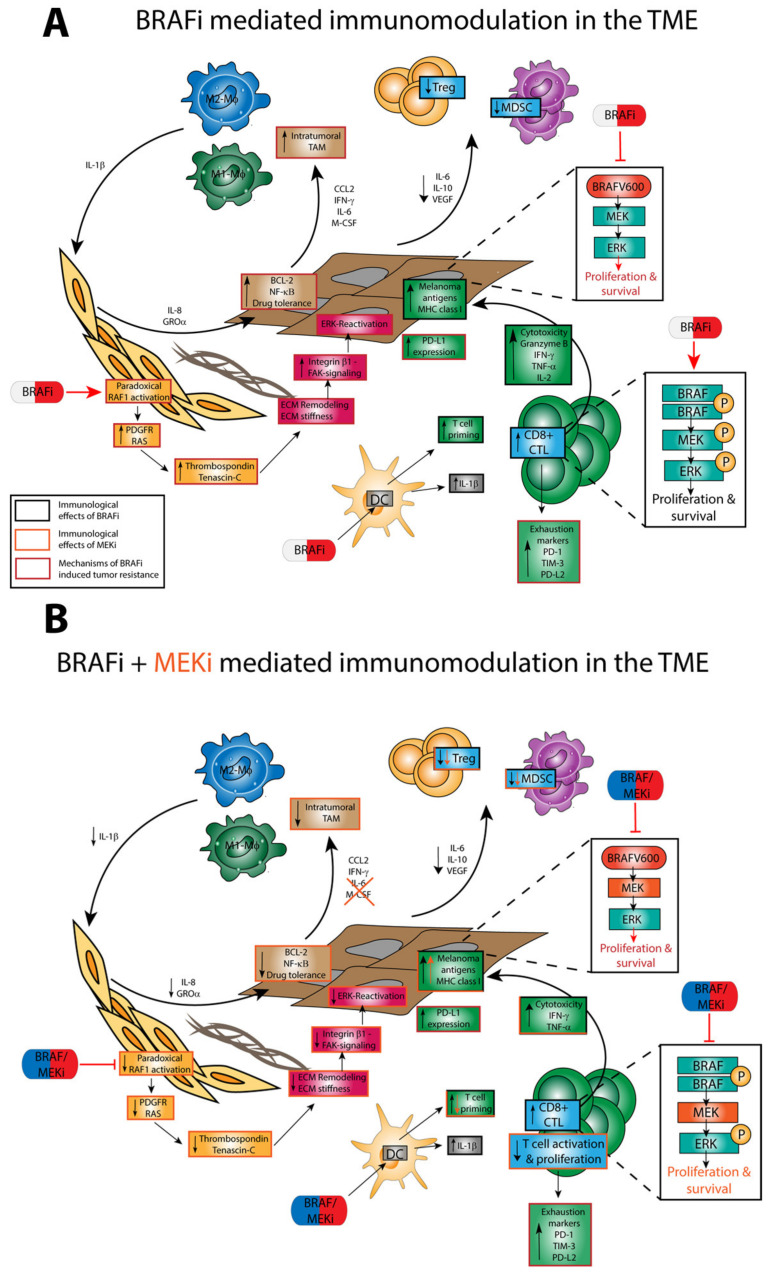
Immunomodulatory effects of BRAF (left) and MEK (right) inhibition on the tumor microenvironment. (**A**) BRAFi exert antiproliferative functions via blockade of ERK signaling in melanoma tumor cells. Moreover, BRAFi exert pivotal immunomodulatory functions within the TME. Here, it has been shown that BRAFi reduce the infiltration of Treg and MDSC within the TME via inhibition of IL-6, IL-10, and VEGF secretion by melanoma cells. Moreover, BRAFi enhance CD8^+^ T cell infiltration and activation via paradoxical ERK activation and augment cytotoxicity in T cells via the secretion of granzyme B, perforin, IFN-γ, and IL-2. Owing to increased immunogenic cell death, the expression of melanoma antigens and MHC-I molecules is significantly enhanced by BRAFi. However, BRAFi monotherapy also results in the development of mechanisms of tumor resistance. Among these, the functional crosstalk between TAM, cancer-associated fibroblasts (CAF), and melanoma cells contribute to rapid resistance to BRAFi [66]. Additionally, CAF contribute to tumor resistance via extracellular matrix (ECM) remodeling, resulting in the reactivation of ERK in melanoma cells [65]. Lastly, it has been found that BRAFi may increase both PD-L1 levels on melanoma cells, which may favor an exhausted T cell phenotype. (**B**) Coadministration of MEKi results in a decreased density of TAM, thus preventing the rapid development of treatment resistance. Further, MEKi prevent the polarization of monocytes into MDSC and the infiltration of regulatory T cells and MDSC into the TME. However, MEKi also reduce the proliferation and activation of T cells via blockade of ERK signaling, thus limiting the T cell stimulatory capacities of BRAFi.

As illustrated in Figure 2a, BRAFi exert off-target effects on immune cells in large part via paradoxical ERK activation [80]. The underlying mechanism of paradoxical ERK activation in somatic cells, which express wild-type BRAF, is presented in more detail below. Co-treatment of melanoma with MEKi was established to overcome resistance mechanisms that reestablished ERK activity [68], and may also counteract paradoxical ERK activation in immune cells. However, due to the general importance of ERK signaling for immune cell functions [81], the application of MEKi may cause (unintended) functional alterations in immune cells as well (Figure 2b).

#### 4.3.1. Paradoxical ERK Activation

Paradoxical ERK activation in response to treatment with second-generation αC-OUT-RAF inhibitors is caused by differences in the modes of action of mutated and wild-type BRAF proteins. BRAF^V600E^ signals downstream as a monomer [82]. Binding of the inhibitor impairs BRAF^V600E^ activity and, thereby, downregulates ERK signaling. In cell types carrying wild-type BRAF, signaling is achieved by homo- and/or heterodimers of BRAF and RAF1 [83]. Binding of BRAFi to a BRAF protein of these dimers locks the enzyme in its active configuration since the inhibitor binds to the ATP-binding site. This change in conformation stimulates activity to the partnering enzyme in the dimer, which initiates downstream signaling, resulting in upregulation of the ERK pathway [82]. The latter causes cell-type-specific side effects such as squamous cell carcinoma in skin cells [84] or changes in the activity of different immune cell types [80], which will be elaborated on below.

#### 4.3.2. TME

Liu et al. investigated the immunological effects of the BRAFi dabrafenib and the MEKi trametinib in a CT26 mouse model [85]. Both drugs induced the expression of apoptosis markers as well as HLA molecules in BRAF^V600^-mutated melanoma cells and decreased the production of anti-inflammatory factors such as PD-L1, IL-1, IL-8, and VEGFA.

Wilmott et al. studied the effects of the BRAFi dabrafenib and vemurafenib on the immune response in tumor biopsies from patients with metastatic malignant melanoma [14]. Tissue samples were collected before and after treatment with BRAFi. Here, a correlation was observed between the extent of tumor infiltration by CD8^+^ T cells and granzyme B-expressing lymphocytes in tumor biopsies taken after BRAFi treatment. In addition, the higher number of CD8^+^ T cells was associated with a smaller tumor size as well as an increase in tumor necrosis [80]. In a similar study, biopsies were taken from patients with metastatic malignant melanoma before and 10–14 days after treatment initiation and were analyzed for melanoma antigens, T cell markers, and immunomodulatory cytokines [86]. Treatment consisted of either vemurafenib monotherapy or combined dabrafenib/trametinib administration. Both treatment strategies correlated with increased expression of melanoma antigens, increased CD8^+^ T cells, and T cell cytotoxicity markers, and decreased levels of the immunosuppressive cytokines IL-6 and IL-8. In addition, inhibitory receptors such as PD-1 and T cell immunoglobulin and mucin-domain containing-3 were increased in expression during treatment. When treated with BRAFi alone, a decrease in melanoma antigen expression and infiltration with CD8^+^ lymphocytes was observed during tumor progression, but this was reversed when combined with a MEKi. The authors conclude from these results that treatment with BRAFi contributes to a TME more favorable for treatment [87].

#### 4.3.3. Regulatory Immune Cells

In a mouse BRAF^V600E^ melanoma model, the administration of dabrafenib increased the number of TAMs and Treg [88]. However, this increase was prevented in the case of combined treatment with dabrafenib and trametinib. Anti-tumor effects were enhanced in response to additional treatment with a PD-1 blocking antibody. In two more studies, BRAFi and MEKi were demonstrated to enhance TAM numbers both in mouse tumor models as well as in human biopsy samples [66,89]. Smith and co-workers demonstrated that TAM generated TNF-α during treatment with BRAF/MEKi, which in turn induced expression of the microphthalmia-associated transcription factor in melanoma cells, preventing cell death [89]. Young and colleagues showed that the elevated number of TAM resulted in exaggerated IL-1α production that, in turn, induced the generation of immunomodulatory soluble mediators in CAF, which evoked expression of anti-apoptotic proteins in tumors cells [66].

Although TAM may consist of both tumor-promoting type-2 macrophages as well as tumor-inhibiting type-1 macrophages, somewhat in contrast to these findings, Schilling et al. found that the number of MDSC in the blood of melanoma patients treated with vemurafenib decreased. The authors hypothesized that vemurafenib inhibited BRAF^V600^-mutated melanoma cells to secrete MDSC-inducing factors, such as IL-6, through immunomodulatory effects [87]. 

#### 4.3.4. DC

Concerning the effects of MAPK-targeting drugs on DC, Ott et al. showed that human monocyte-derived (MO-)DC, when stimulated and subsequently co-incubated with BRAF^V600E^-mutated melanoma cell lines, displayed impaired expression of costimulatory receptors and cytokine production [44]. These effects were reversed in the presence of vemurafenib and/or a MEKi. Direct administration of the MEKi to DC resulted in pronounced inhibition of viability and T cell priming capacity. Vemurafenib, on the other hand, showed no effect on DC functions over a wide range of concentrations. The authors concluded that BRAF^V600E^-mutated melanoma cells suppress DC function, but that this can be restored by blocking the ERK pathway.

Tel et al. also investigated the effect of vemurafenib on the functional capacity of DC. For this purpose, plasmacytoid (p)DC and conventional (c)DC were isolated from PBMC provided by healthy donors or from melanoma patients treated with vemurafenib [90]. Maturation of either DC population by the TLR-7/8 ligand R848 was inhibited in case of concomitant treatment with vemurafenib, both in terms of surface maturation marker expression and cytokine production, leading to decreased allogeneic T cell proliferation. Interestingly, the inhibitory effect of vemurafenib on DC activation was not observed when total PBMC were treated. The authors suggested that the presence of other cell types resulted in decreased uptake of vemurafenib by DC, thereby preventing inhibitory effects.

In another study that issued potential effects of the BRAFi dabrafenib, no modulatory effects on the immunophenotype of human MO-DC and stimulated T cells were observed [91]. In contrast, the MEKi trametinib, when applied in combination with dabrafenib, suppressed the antigen cross-presenting activity of MO-DC. Furthermore, the application of trametinib alone or in combination with dabrafenib attenuated T cell proliferation.

Our lab investigated off-target effects of vemurafenib and dabrafenib, and of the according MEKi cobimetinib and trametinib on the immunophenotype of murine DC [15]. We observed that the administration of dabrafenib to murine bone marrow-derived or splenic DC increased the expression of MHC-II, of the costimulatory receptors (CD80, CD86) and increased IL-1β production. Besides enhancing IL-1β mRNA expression, dabrafenib also activated Caspase-1, which confers cleavage of pro-IL-1β to yield bioactive IL-1β [17]. Moreover, dabrafenib applied at high concentrations also activated Caspase-8, which cleaves pro-IL-1β as well. In contrast, vemurafenib was less potent with regard to DC activation and conferred IL-1β production only when co-applied with LPS. Trametinib and cobimetinib did not counteract the effects of either BRAFi, which suggested that the DC stimulatory properties were not due to paradoxical ERK activation. Similar effects were also noted for human MO-DC, with vemurafenib being more potent than dabrafenib. 

These results are of considerable interest in light of the results of a very extensive study from Young and co-workers, which demonstrated an increase in IL-1β expression in tumor biopsies of patients under treatment with vemurafenib or a combination of dabrafenib and trametinib [66]. The elevated IL-1β levels were attributed to TAM, which was apparent at a higher frequency in the tumors of patients treated with BRAFi/MEKi. TAM-derived IL-1β was demonstrated to upregulate expression of IL-8 and the chemokine C–X–C motif ligand 1 in CAF, which in turn increased tumor survival by mediating upregulation of anti-apoptotic proteins in tumor cells. The pro-tumorigenic properties of IL-1β were confirmed in vivo using IL-1 receptor-deficient mice, which, when inoculated with BRAF^V600E^ melanoma cells, showed attenuated tumor growth as compared to wild-type mice. Likewise, clinical administration of the anti-IL-1 antibody canakinumab significantly reduced the occurrence of lung cancers in a clinical trial with more than 15,000 patients (Cantos), suggesting that IL-1 may have tumor-promoting properties. However, a follow-up trial that combined canakinumab with docetaxel (Canopy-2) failed to enhance response rates compared to chemotherapy alone. A trial with anti-IL-1 plus anti-PD1 (Chorus) in non-small cell lung cancer is still ongoing.

More recently, Riegel and colleagues reported that the pan-RAF inhibitor LY3009120, which in contrast to second-generation αC-OUT-RAF inhibitors does not cause paradoxical ERK activation [92], interfered with the activation of murine bone marrow-derived DC and human MO-DC, in contrast to the MEKi trametinib [93]. The authors proposed a ‘non linear MAPK pathway’ [94] in DC, meaning that RAF and MEK kinases may exert distinct roles in DC biology. This is in contrast to the situation in T cells where the pan-RAF inhibitor and MEK inhibitors have similar effects [93].

#### 4.3.5. T Cells

It has been reported that in melanoma patients, monotherapy with vemurafenib, but not dabrafenib, decreased peripheral lymphocyte numbers [95]. Within the CD4^+^ T cell compartment, a decrease in central memory cells, but an increase in naïve T cells, was observed. In addition, secretion of IFN-γ and IL-9 by CD4^+^ T cells was significantly lower in vemurafenib-treated samples than measured in pre-treatment samples. Callahan and co-workers investigated the effects of the pan-RAF inhibitor BMS908662 on T cell activation [80]. They found that T cell activation increased in a RAFi concentration-dependent manner and that this effect correlated with enhanced ERK signaling based on paradoxical activation of ERK by the RAFi. Furthermore, proliferation of polyclonally activated CD4^+^ (up to 25%) and CD8^+^ (up to 53%) T cells was increased by the RAFi [15].

Liu and co-workers assessed the immunological effects of dabrafenib and trametinib in vitro in human CD4^+^ and CD8^+^ T cells obtained from healthy donors [85]. They demonstrated that dabrafenib increased phospho-ERK expression in human T cells and left CD4^+^ and CD8^+^ T cell function unchanged, whereas trametinib decreased pERK levels and led to partial or transient inhibition of T cell proliferation and alteration of cytokine production. The effects of trametinib were partially attenuated by the addition of dabrafenib.

The result of MEK inhibition may depend on the activation state of the T cell when it encounters an according inhibitor. As shown by Ebert and co-workers in a mouse colon carcinoma model, the MEKi G-38963 (similar to cobimetinib) increased the number of tumor-infiltrating CD8^+^ T cells, associated with decreased tumor growth, and these effects were enhanced when the MEKi and a PD-L1 blocking antibody were co-administered [96]. The authors attributed the outcome to a protective effect of MEK inhibition for the tumor-infiltrating T cells against exhaustive T cell death (as they are already active before MEKi treatment). In contrast, MEKi were found to inhibit the priming of naïve T cells. Furthermore, it was shown that MEK activity is not essential for CTL effector functions in vitro. Beneficial effects on T cell function were also discovered in a pulsatile form of MEK inhibition treatment [97]. This was seen especially in CD8^+^ T cells. These showed increased infiltration in a non-small-cell lung carcinoma tumor model and a higher expression of Ki-67, as compared to continuous treatment. The pulsatile treatment showed the longest PFS in mice and the combination of pulsatile treatment and anti-CTLA-4 antibody resulted in the longest OS. These findings suggest that it may be interesting to reevaluate the dosing schedule of MEKi treatments to maximize their therapeutic benefits.

In general, there seems to be little scientific evidence on BRAF/MEK inhibition regarding differentiated T cell populations in tumor-bearing mice or humans. It warrants further study to examine if the effects of targeted therapy on T cells differ dependent on the activation state of either T cell subpopulation when encountered by the inhibitor.

## 5. Conclusions/Perspectives

The introduction of MAPK-targeted therapeutics has considerably improved the treatment options for patients bearing BRAF-mutated melanoma [4,12]. Preclinical data and clinical analysis of human tumor material have demonstrated that MAPK-targeted therapy may alter TME conditions early after treatment induction by decreasing the levels of immunosuppressive cytokines and PD-1/PD-L1 expression levels, thereby increasing effector T cell infiltration [14,88,98,99,100,101]. These immunological changes may help to overcome treatment resistance to ICI and augment anti-tumor responses driven by checkpoint blockade. In agreement, several clinical trials on BRAF-mutated melanoma have confirmed the efficacy and tolerability of co-administered BRAFi/MEKi and ICI (e.g., NCT02130466 [102]; NCT02967692 [103]). Further, in a phase I clinical trial co-administration of the BRAFi dabrafenib, the according MEKi cobimetinib, and a PD-L1 specific antibody (durvalumab) yielded a higher ORR in patients suffering from BRAF-mutated metastatic melanoma as compared to the application of the MEKi and the ICI alone [104]. Interestingly, in a clinical phase III study, co-treatment of patients with BRAF wild-type melanoma with a MEKi (cobimetinib) and the PD-L1 inhibitor atezolizumab also resulted in a longer PFS as compared to monotherapy with the PD-1 specific antibody Pembrolizumab, and resulted in a higher rate of AE (NCT03273153) [105].

Furthermore, it has been shown that the favorable immune effects mediated by BRAF/MEKi are paralleled by the induction of T cell exhaustion markers and, thus, treatment response appears to subside in the long-term [100]. Therefore, it has been proposed by a number of preclinical studies and clinical trials that the efficacy of ICI [106] and BRAFi/MEKi [19,107] may be considerably enhanced by co-administration of additional anti-tumor drugs (such as conventional chemo or radiotherapy), as well as agents which act on regulatory immune cells (i.e., antiangiogenic drugs, tyrosine kinase receptor antagonists).

However, as discussed in this review, unexpected effects of therapeutic MAPK inhibition on (immune) cells within the TME and the periphery [81,108] may considerably affect the overall efficacy of treatment, which warrants further studies. More specifically, research should continue to monitor the impact of applied inhibitors at different stages of illness and treatment on different immune cell populations very closely. Insight into the response of these cells to treatment might be of utmost importance to understand the therapeutic results and the observed side effects of the respective treatment. This may benefit the rational development of inhibitors and combination therapies, and might also enable the induction of profound and sustained anti-tumor responses in non-responders. The aspect of unintended modulation of immune cell functions should also become an issue when assessing the suitability of currently developed RAF inhibitors [75] in order to maximize therapeutic efficacies.

## Abbreviations

AE	Adverse event
APC	Antigen-presenting cell
BCL	B-Cell chronic lymphocytic leukemia/lymphoma
BRAF	v-Raf murine sarcoma viral oncogene homolog B
BRAFi	BRAF inhibitors (BRAFi)
CAF	Cancer-associated fibroblast
cDC	Conventional DC
CDK	Cyclin-dependent kinase
CDKN	Cyclin-dependent kinase inhibitor
CTLA-4	Cytotoxic T-lymphocyte associated protein
DC	Dendritic cell
ERK	Extracellular signal-regulated kinase
GTP	Guanosine 5’-triphosphate
HGF	Hepatocyte growth factor
ICI	Immune checkpoint inhibitor
IFN	Interferon
IL	Interleukin
LPS	Lipopolysaccharide
MAPK	Mitogen-activated protein kinase
MDSC	Myeloid-derived suppressor cell
MEK	MAPK/ERK kinase
MEKi	MEK inhibitor
MHC	Major histocompatibility complex
MMP	Matrix metalloprotease
MO-DC	Monocyte-derived DC
NF1	Neurofibroma 1
NRAS	Neuroblastoma RAS viral oncogene homolog
PBMC	Peripheral blood mononuclear cells
PD-1	Programmed cell death
PD-L1	PD-1 ligand 1
pDC	Plasmacytoid DC
PI3K	Phosphoinositide 3-kinases
PLCγ	Phospholipase Cγ
PFS	Progression-free survival
PTEN	Phosphatase and tensin homolog
RAF	Rat fibrosarcoma
RAS	Rat sarcoma
RTK	Receptor tyrosine kinase
siRNA	Silencer RNA
STAT	Signal transducer and activator of transcription
TAM	Tumor-associated macrophage
TERT	Telomerase reverse transcriptase
TIL	Tumor-infiltrating leukocyte
TNF	Tumor necrosis factor
TLRT	Toll-like receptor
TME	Tumor microenvironment
Treg	Regulatory T cell
VEGF	Vascular endothelial growth factor

## References

[1] Leiter U., Keim U., Garbe C. (2020). Epidemiology of Skin Cancer: Update 2019. Adv. Exp. Med. Biol..

[2] Schadendorf D., Hodi F.S., Robert C., Weber J.S., Margolin K., Hamid O., Patt D., Chen T.-T., Berman D.M., Wolchok J.D. (2015). Pooled Analysis of Long-Term Survival Data From Phase II and Phase III Trials of Ipilimumab in Unresectable or Metastatic Melanoma. J. Clin. Oncol..

[3] Franklin C., Livingstone E., Roesch A., Schilling B., Schadendorf D. (2017). Immunotherapy in melanoma: Recent advances and future directions. Eur. J. Surg. Oncol..

[4] Singh S., Numan A., Agrawal N., Tambuwala M.M., Singh V., Kesharwani P. (2020). Role of immune checkpoint inhibitors in the revolutionization of advanced melanoma care. Int. Immunopharmacol..

[5] Hamid O., Robert C., Daud A., Hodi F.S., Hwu W.J., Kefford R., Wolchok J.D., Hersey P., Joseph R., Weber J.S. (2019). Five-year survival outcomes for patients with advanced melanoma treated with pembrolizumab in KEYNOTE-001. Ann. Oncol..

[6] Czarnecka A.M., Bartnik E., Fiedorowicz M., Rutkowski P. (2020). Targeted Therapy in Melanoma and Mechanisms of Resistance. Int. J. Mol. Sci..

[7] Savoia P., Fava P., Casoni F., Cremona O. (2019). Targeting the ERK Signaling Pathway in Melanoma. Int. J. Mol. Sci..

[8] Flaherty K.T., Puzanov I., Kim K.B., Ribas A., McArthur G.A., Sosman J.A., O’Dwyer P.J., Lee R.J., Grippo J.F., Nolop K. (2010). Inhibition of mutated, activated BRAF in metastatic melanoma. N. Engl. J. Med..

[9] Degirmenci U., Wang M., Hu J. (2020). Targeting Aberrant RAS/RAF/MEK/ERK Signaling for Cancer Therapy. Cells.

[10] Proietti I., Skroza N., Michelini S., Mambrin A., Balduzzi V., Bernardini N., Marchesiello A., Tolino E., Volpe S., Maddalena P. (2020). BRAF Inhibitors: Molecular Targeting and Immunomodulatory Actions. Cancers.

[11] Aplin A.E., Kaplan F.M., Shao Y. (2011). Mechanisms of resistance to RAF inhibitors in melanoma. J. Invest. Dermatol..

[12] Ottaviano M., Giunta E.F., Tortora M., Curvietto M., Attademo L., Bosso D., Cardalesi C., Rosanova M., De Placido P., Pietroluongo E. (2021). *BRAF* Gene and Melanoma: Back to the Future. Int. J. Mol. Sci..

[13] Flaherty K.T., Robert C., Hersey P., Nathan P., Garbe C., Milhem M., Demidov L.V., Hassel J.C., Rutkowski P., Mohr P. (2012). Improved survival with MEK inhibition in BRAF-mutated melanoma. N. Engl. J. Med..

[14] Wilmott J.S., Long G.V., Howle J.R., Haydu L.E., Sharma R.N., Thompson J.F., Kefford R.F., Hersey P., Scolyer R.A. (2012). Selective BRAF inhibitors induce marked T-cell infiltration into human metastatic melanoma. Clin. Cancer Res..

[15] Hajek E., Krebs F., Bent R., Haas K., Bast A., Steinmetz I., Tuettenberg A., Grabbe S., Bros M. (2018). BRAF inhibitors stimulate inflammasome activation and interleukin 1 beta production in dendritic cells. Oncotarget.

[16] Karki R., Kanneganti T.D. (2019). Diverging inflammasome signals in tumorigenesis and potential targeting. Nat. Rev. Cancer.

[17] Bent R., Moll L., Grabbe S., Bros M. (2018). Interleukin-1 Beta-A Friend or Foe in Malignancies?. Int. J. Mol. Sci..

[18] Zhang M., Bhat T., Gutmann D.H., Johnson K.J. (2019). Melanoma in individuals with neurofibromatosis type 1: A retrospective study. Dermatol. Online J..

[19] Delyon J., Lebbe C., Dumaz N. (2020). Targeted therapies in melanoma beyond BRAF: Targeting NRAS-mutated and KIT-mutated melanoma. Curr. Opin. Oncol..

[20] Shain A.H., Bastian B.C. (2016). From melanocytes to melanomas. Nat. Rev. Cancer.

[21] Dong J., Phelps R.G., Qiao R., Yao S., Benard O., Ronai Z., Aaronson S.A. (2003). BRAF oncogenic mutations correlate with progression rather than initiation of human melanoma. Cancer Res..

[22] Liang W.S., Hendricks W., Kiefer J., Schmidt J., Sekar S., Carpten J., Craig D.W., Adkins J., Cuyugan L., Manojlovic Z. (2017). Integrated genomic analyses reveal frequent *TERT* aberrations in acral melanoma. Genome Res..

[23] Hernando B., Swope V.B., Guard S., Starner R.J., Choi K., Anwar A., Cassidy P., Leachman S., Kadekaro A.L., Bennett D.C. (2019). *In vitro* behavior and UV response of melanocytes derived from carriers of *CDKN2A* mutations and *MC1R* variants. Pigment Cell Melanoma Res..

[24] Mandal R., Becker S., Strebhardt K. (2016). Stamping out RAF and MEK1/2 to inhibit the ERK1/2 pathway: An emerging threat to anticancer therapy. Oncogene.

[25] Śmiech M., Leszczyński P., Kono H., Wardell C., Taniguchi H. (2020). Emerging *BRAF* Mutations in Cancer Progression and Their Possible Effects on Transcriptional Networks. Genes.

[26] Cheng L., Lopez-Beltran A., Massari F., MacLennan G.T., Montironi R. (2018). Molecular testing for *BRAF* mutations to inform melanoma treatment decisions: A move toward precision medicine. Mod. Pathol..

[27] Wan P.T., Garnett M.J., Roe S.M., Lee S., Niculescu-Duvaz D., Good V.M., Jones C.M., Marshall C.J., Springer C.J., Barford D. (2004). Mechanism of activation of the RAF-ERK signaling pathway by oncogenic mutations of B-RAF. Cell.

[28] Muñoz-Couselo E., Adelantado E.Z., Ortiz C., García J.S., Perez-Garcia J. (2017). NRAS-mutant melanoma: Current challenges and future prospect. Oncotargets Ther..

[29] Sensi M., Nicolini G., Petti C., Bersani I., Lozupone F., Molla A., Vegetti C., Nonaka D., Mortarini R., Parmiani G. (2006). Mutually exclusive NRASQ61R and BRAFV600E mutations at the single-cell level in the same human melanoma. Oncogene.

[30] Krauthammer M., Kong Y., Bacchiocchi A., Evans P., Pornputtapong N., Wu C., McCusker J.P., Ma S., Cheng E., Straub R. (2015). Exome sequencing identifies recurrent mutations in *NF1* and *RAS* opathy genes in sun-exposed melanomas. Nat. Genet..

[31] Kiuru M., Busam K.J. (2017). The *NF1* gene in tumor syndromes and melanoma. Lab. Investig..

[32] Nissan M.H., Pratilas C.A., Jones A.M., Ramirez R., Won H., Liu C., Tiwari S., Kong L., Hanrahan A.J., Yao Z. (2014). Loss of NF1 in cutaneous melanoma is associated with RAS activation and MEK dependence. Cancer Res..

[33] Shain A.H., Yeh I., Kovalyshyn I., Sriharan A., Talevich E., Gagnon A., Dummer R., North J., Pincus L., Ruben B. (2015). The Genetic Evolution of Melanoma from Precursor Lesions. N. Engl. J. Med..

[34] Smith S.L., Pitt A.R., Spickett C.M. (2021). Approaches to Investigating the Protein Interactome of PTEN. J. Proteome Res..

[35] Aguissa-Touré A.H., Li G. (2012). Genetic alterations of *PTEN* in human melanoma. Cell. Mol. Life Sci..

[36] Hilke F.J., Sinnberg T., Gschwind A., Niessner H., Demidov G., Amaral T., Ossowski S., Bonzheim I., Röcken M., Riess O. (2020). Distinct Mutation Patterns Reveal Melanoma Subtypes and Influence Immunotherapy Response in Advanced Melanoma Patients. Cancers.

[37] Dankort D., Curley D.P., Cartlidge R.A., Nelson B., Karnezis A.N., Damsky W.E., You M.J., DePinho R.A., McMahon M., Bosenberg M. (2009). *Braf*(*V600E*) cooperates with *Pten* loss to induce metastatic melanoma. Nat. Genet..

[38] Bradley S.D., Chen Z., Melendez B., Talukder A., Khalili J.S., Rodriguez-Cruz T., Liu S., Whittington M., Deng W., Li F. (2015). BRAF^V600E^ Co-opts a Conserved MHC Class I Internalization Pathway to Diminish Antigen Presentation and CD8^+^ T-cell Recognition of Melanoma. Cancer Immunol. Res..

[39] Shabaneh T.B., Molodtsov A.K., Steinberg S.M., Zhang P., Torres G.M., Mohamed G.A., Boni A., Curiel T.J., Angeles C.V., Turk M.J. (2018). Oncogenic BRAF(V600E) Governs Regulatory T-cell Recruitment during Melanoma Tumorigenesis. Cancer Res..

[40] Ho P.-C., Meeth K.M., Tsui Y.-C., Srivastava B., Bosenberg M.W., Kaech S.M. (2014). Immune-Based Antitumor Effects of BRAF Inhibitors Rely on Signaling by CD40L and IFNγ. Cancer Res..

[41] Leslie C., Bowyer S.E., White A., Grieu-Iacopetta F., Trevenen M., Iacopetta B., Amanuel B., Millward M. (2015). FOXP3+ T regulatory lymphocytes in primary melanoma are associated with BRAF mutation but not with response to BRAF inhibitor. Pathology.

[42] Kuske M., Westphal D., Wehner R., Schmitz M., Beissert S., Praetorius C., Meier F. (2018). Immunomodulatory effects of BRAF and MEK inhibitors: Implications for Melanoma therapy. Pharmacol. Res..

[43] Sumimoto H., Imabayashi F., Iwata T., Kawakami Y. (2006). The BRAF-MAPK signaling pathway is essential for cancer-immune evasion in human melanoma cells. J. Exp. Med..

[44] Ott P.A., Henry T., Baranda S.J., Frleta D., Manches O., Bogunovic D., Bhardwaj N. (2013). Inhibition of both BRAF and MEK in BRAF(V600E) mutant melanoma restores compromised dendritic cell (DC) function while having differential direct effects on DC properties. Cancer Immunol. Immunother..

[45] Kim K., Jeon S., Kim T.M., Jung C.K. (2018). Immune Gene Signature Delineates a Subclass of Papillary Thyroid Cancer with Unfavorable Clinical Outcomes. Cancers.

[46] Khalili J.S., Liu S., Rodríguez-Cruz T.G., Whittington M., Wardell S., Liu C., Zhang M., Cooper Z.A., Frederick D.T., Li Y. (2012). Oncogenic BRAF(V600E) promotes stromal cell-mediated immunosuppression via induction of interleukin-1 in melanoma. Clin. Cancer Res..

[47] Binnewies M., Roberts E.W., Kersten K., Chan V., Fearon D.F., Merad M., Coussens L.M., Gabrilovich D.I., Ostrand-Rosenberg S., Hedrick C.C. (2018). Understanding the tumor immune microenvironment (TIME) for effective therapy. Nat. Med..

[48] Whipple C.A., Boni A., Fisher J.L., Hampton T.H., Tsongalis G.J., Mellinger D.L., Yan S., Tafe L.J., Brinckerhoff C.E., Turk M.J. (2016). The mitogen-activated protein kinase pathway plays a critical role in regulating immunological properties of BRAF mutant cutaneous melanoma cells. Melanoma Res..

[49] Terai M., Eto M., Young G.D., Berd D., Mastrangelo M.J., Tamura Y., Harigaya K., Sato T. (2012). Interleukin 6 mediates production of interleukin 10 in metastatic melanoma. Cancer Immunol. Immunother..

[50] Caporali S., Alvino E., Lacal P.M., Levati L., Giurato G., Memoli D., Caprini E., Antonini Cappellini G.C., D’Atri S. (2016). Targeting the PI3K/AKT/mTOR pathway overcomes the stimulating effect of dabrafenib on the invasive behavior of melanoma cells with acquired resistance to the BRAF inhibitor. Int. J. Oncol..

[51] Dowling J., McGregor S.P., Williford P. (2019). Update on Current Treatment Recommendations for Primary Cutaneous Melanoma. Dermatol. Clin..

[52] Corrie P., Hategan M., Fife K., Parkinson C. (2014). Management of melanoma. Br. Med. Bull..

[53] Sullivan R.J. (2018). The role of targeted therapy for melanoma in the immunotherapy era. Semin. Cutan. Med. Surg..

[54] Ny L., Hernberg M., Nyakas M., Koivunen J., Oddershede L., Yoon M., Wang X., Guyot P., Geisler J. (2020). *BRAF* mutational status as a prognostic marker for survival in malignant melanoma: A systematic review and meta-analysis. Acta Oncol..

[55] Patel H., Yacoub N., Mishra R., White A., Long Y., Alanazi S., Garrett J.T. (2020). Current Advances in the Treatment of BRAF-Mutant Melanoma. Cancers.

[56] Su F., Viros A., Milagre C., Trunzer K., Bollag G., Spleiss O., Reis-Filho J.S., Kong X., Koya R.C., Flaherty K.T. (2012). RAS mutations in cutaneous squamous-cell carcinomas in patients treated with BRAF inhibitors. N. Engl. J. Med..

[57] Manzano J.L., Layos L., Bugés C., de Los Llanos Gil M., Vila L., Martínez-Balibrea E., Martínez-Cardús A. (2016). Resistant mechanisms to BRAF inhibitors in melanoma. Ann. Transl. Med..

[58] Nazarian R., Shi H., Wang Q., Kong X., Koya R.C., Lee H., Chen Z., Lee M.K., Attar N., Sazegar H. (2010). Melanomas acquire resistance to B-RAF(V600E) inhibition by RTK or N-RAS upregulation. Nature.

[59] Lito P., Pratilas C.A., Joseph E.W., Tadi M., Halilovic E., Zubrowski M., Huang A., Wong W.L., Callahan M.K., Merghoub T. (2012). Relief of profound feedback inhibition of mitogenic signaling by RAF inhibitors attenuates their activity in BRAFV600E melanomas. Cancer Cell.

[60] Poulikakos P.I., Persaud Y., Janakiraman M., Kong X., Ng C., Moriceau G., Shi H., Atefi M., Titz B., Gabay M.T. (2011). RAF inhibitor resistance is mediated by dimerization of aberrantly spliced BRAF(V600E). Nature.

[61] Emery C.M., Vijayendran K.G., Zipser M.C., Sawyer A.M., Niu L., Kim J.J., Hatton C., Chopra R., Oberholzer P.A., Karpova M.B. (2009). MEK1 mutations confer resistance to MEK and B-RAF inhibition. Proc. Natl. Acad. Sci. USA.

[62] Wagle N., Van Allen E.M., Treacy D.J., Frederick D.T., Cooper Z.A., Taylor-Weiner A., Rosenberg M., Goetz E.M., Sullivan R.J., Farlow D.N. (2014). MAP kinase pathway alterations in BRAF-mutant melanoma patients with acquired resistance to combined RAF/MEK inhibition. Cancer Discov..

[63] Dratkiewicz E., Simiczyjew A., Mazurkiewicz J., Ziętek M., Matkowski R., Nowak D. (2021). Hypoxia and Extracellular Acidification as Drivers of Melanoma Progression and Drug Resistance. Cells.

[64] Straussman R., Morikawa T., Shee K., Barzily-Rokni M., Qian Z.R., Du J., Davis A., Mongare M.M., Gould J., Frederick D.T. (2012). Tumour micro-environment elicits innate resistance to RAF inhibitors through HGF secretion. Nature.

[65] Hirata E., Girotti M.R., Viros A., Hooper S., Spencer-Dene B., Matsuda M., Larkin J., Marais R., Sahai E. (2015). Intravital imaging reveals how BRAF inhibition generates drug-tolerant microenvironments with high integrin β1/FAK signaling. Cancer Cell.

[66] Young H.L., Rowling E.J., Bugatti M., Giurisato E., Luheshi N., Arozarena I., Acosta J.C., Kamarashev J., Frederick D.T., Cooper Z.A. (2017). An adaptive signaling network in melanoma inflammatory niches confers tolerance to MAPK signaling inhibition. J. Exp. Med..

[67] Wang T., Xiao M., Ge Y., Krepler C., Belser E., Lopez-Coral A., Xu X., Zhang G., Azuma R., Liu Q. (2015). BRAF Inhibition Stimulates Melanoma-Associated Macrophages to Drive Tumor Growth. Clin. Cancer Res..

[68] Arozarena I., Wellbrock C. (2017). Overcoming resistance to BRAF inhibitors. Ann. Transl. Med..

[69] Ascierto P.A., McArthur G.A., Dréno B., Atkinson V., Liszkay G., Di Giacomo A.M., Mandalà M., Demidov L., Stroyakovskiy D., Thomas L. (2016). Cobimetinib combined with vemurafenib in advanced BRAF(V600)-mutant melanoma (coBRIM): Updated efficacy results from a randomised, double-blind, phase 3 trial. Lancet Oncol..

[70] Spain L., Julve M., Larkin J. (2016). Combination dabrafenib and trametinib in the management of advanced melanoma with BRAFV600 mutations. Expert. Opin. Pharmacother..

[71] Ascierto P.A., Dummer R., Gogas H.J., Flaherty K.T., Arance A., Mandala M., Liszkay G., Garbe C., Schadendorf D., Krajsova I. (2020). Update on tolerability and overall survival in COLUMBUS: Landmark analysis of a randomised phase 3 trial of encorafenib plus binimetinib vs vemurafenib or encorafenib in patients with BRAF V600-mutant melanoma. Eur. J. Cancer.

[72] Moriceau G., Hugo W., Hong A., Shi H., Kong X., Yu C.C., Koya R.C., Samatar A.A., Khanlou N., Braun J. (2015). Tunable-combinatorial mechanisms of acquired resistance limit the efficacy of BRAF/MEK cotargeting but result in melanoma drug addiction. Cancer Cell.

[73] Lu H., Liu S., Zhang G., Bin W., Zhu Y., Frederick D.T., Hu Y., Zhong W., Randell S., Sadek N. (2017). PAK signalling drives acquired drug resistance to MAPK inhibitors in BRAF-mutant melanomas. Nature.

[74] Broman K.K., Dossett L.A., Sun J., Eroglu Z., Zager J.S. (2019). Update on BRAF and MEK inhibition for treatment of melanoma in metastatic, unresectable, and adjuvant settings. Expert Opin. Drug Saf..

[75] Dumaz N., Lebbé C. (2021). New perspectives on targeting RAF, MEK and ERK in melanoma. Curr. Opin. Oncol..

[76] Jin T., Lavoie H., Sahmi M., David M., Hilt C., Hammell A., Therrien M. (2017). RAF inhibitors promote RAS-RAF interaction by allosterically disrupting RAF autoinhibition. Nat. Commun..

[77] Karoulia Z., Wu Y., Ahmed T.A., Xin Q., Bollard J., Krepler C., Wu X., Zhang C., Bollag G., Herlyn M. (2016). An Integrated Model of RAF Inhibitor Action Predicts Inhibitor Activity against Oncogenic BRAF Signaling. Cancer Cell.

[78] Karoulia Z., Gavathiotis E., Poulikakos P.I. (2017). New perspectives for targeting RAF kinase in human cancer. Nat. Rev. Cancer.

[79] Mukai K., Kamata M., Miyazaki M., Nagata M., Fukaya S., Hayashi K., Fukuyasu A., Ishikawa T., Ohnishi T., Tada Y. (2021). Edoxaban prevented adverse effects including pyrexia and elevation of D-dimer caused by the combination of BRAF and MEK inhibitors in a patient with BRAF-mutant melanoma. J. Dermatol..

[80] Callahan M.K., Masters G., Pratilas C.A., Ariyan C., Katz J., Kitano S., Russell V., Gordon R.A., Vyas S., Yuan J. (2014). Paradoxical activation of T cells via augmented ERK signaling mediated by a RAF inhibitor. Cancer Immunol. Res..

[81] Arthur J.S., Ley S.C. (2013). Mitogen-activated protein kinases in innate immunity. Nat. Rev. Immunol..

[82] Poulikakos P.I., Zhang C., Bollag G., Shokat K.M., Rosen N. (2010). RAF inhibitors transactivate RAF dimers and ERK signalling in cells with wild-type BRAF. Nature.

[83] Heidorn S.J., Milagre C., Whittaker S., Nourry A., Niculescu-Duvas I., Dhomen N., Hussain J., Reis-Filho J.S., Springer C.J., Pritchard C. (2010). Kinase-dead BRAF and oncogenic RAS cooperate to drive tumor progression through CRAF. Cell.

[84] Peng L., Wang Y., Hong Y., Ye X., Shi P., Zhang J., Zhao Q. (2017). Incidence and relative risk of cutaneous squamous cell carcinoma with single-agent BRAF inhibitor and dual BRAF/MEK inhibitors in cancer patients: A meta-analysis. Oncotarget.

[85] Liu L., Mayes P.A., Eastman S., Shi H., Yadavilli S., Zhang T., Yang J., Seestaller-Wehr L., Zhang S.Y., Hopson C. (2015). The BRAF and MEK Inhibitors Dabrafenib and Trametinib: Effects on Immune Function and in Combination with Immunomodulatory Antibodies Targeting PD-1, PD-L1, and CTLA-4. Clin. Cancer Res..

[86] Frederick D.T., Piris A., Cogdill A.P., Cooper Z.A., Lezcano C., Ferrone C.R., Mitra D., Boni A., Newton L.P., Liu C. (2013). BRAF inhibition is associated with enhanced melanoma antigen expression and a more favorable tumor microenvironment in patients with metastatic melanoma. Clin. Cancer Res..

[87] Schilling B., Paschen A. (2013). Immunological consequences of selective BRAF inhibitors in malignant melanoma: Neutralization of myeloid-derived suppressor cells. Oncoimmunology.

[88] Hu-Lieskovan S., Mok S., Homet Moreno B., Tsoi J., Robert L., Goedert L., Pinheiro E.M., Koya R.C., Graeber T.G., Comin-Anduix B. (2015). Improved antitumor activity of immunotherapy with BRAF and MEK inhibitors in BRAF(V600E) melanoma. Sci. Transl. Med..

[89] Smith M.P., Sanchez-Laorden B., O’Brien K., Brunton H., Ferguson J., Young H., Dhomen N., Flaherty K.T., Frederick D.T., Cooper Z.A. (2014). The immune microenvironment confers resistance to MAPK pathway inhibitors through macrophage-derived TNFα. Cancer Discov..

[90] Tel J., Koornstra R., de Haas N., van Deutekom V., Westdorp H., Boudewijns S., van Erp N., Di Blasio S., Gerritsen W., Figdor C.G. (2016). Preclinical exploration of combining plasmacytoid and myeloid dendritic cell vaccination with BRAF inhibition. J. Transl. Med..

[91] Vella L.J., Pasam A., Dimopoulos N., Andrews M., Knights A., Puaux A.L., Louahed J., Chen W., Woods K., Cebon J.S. (2014). MEK inhibition, alone or in combination with BRAF inhibition, affects multiple functions of isolated normal human lymphocytes and dendritic cells. Cancer Immunol. Res..

[92] Peng S.B., Henry J.R., Kaufman M.D., Lu W.P., Smith B.D., Vogeti S., Rutkoski T.J., Wise S., Chun L., Zhang Y. (2015). Inhibition of RAF Isoforms and Active Dimers by LY3009120 Leads to Anti-tumor Activities in RAS or BRAF Mutant Cancers. Cancer Cell.

[93] Riegel K., Schlöder J., Sobczak M., Jonuleit H., Thiede B., Schild H., Rajalingam K. (2020). RAF kinases are stabilized and required for dendritic cell differentiation and function. Cell Death Differ..

[94] Riegel K., Rajalingam K. (2020). The non-linearity of RAF-MEK signaling in dendritic cells. Cell Cycle.

[95] Schilling B., Sondermann W., Zhao F., Griewank K.G., Livingstone E., Sucker A., Zelba H., Weide B., Trefzer U., Wilhelm T. (2014). Differential influence of vemurafenib and dabrafenib on patients’ lymphocytes despite similar clinical efficacy in melanoma. Ann. Oncol..

[96] Ebert P.J.R., Cheung J., Yang Y., McNamara E., Hong R., Moskalenko M., Gould S.E., Maecker H., Irving B.A., Kim J.M. (2016). MAP Kinase Inhibition Promotes T Cell and Anti-tumor Activity in Combination with PD-L1 Checkpoint Blockade. Immunity.

[97] Choi H., Deng J., Li S., Silk T., Dong L., Brea E.J., Houghton S., Redmond D., Zhong H., Boiarsky J. (2019). Pulsatile MEK Inhibition Improves Anti-tumor Immunity and T Cell Function in Murine Kras Mutant Lung Cancer. Cell Rep..

[98] Mandalà M., De Logu F., Merelli B., Nassini R., Massi D. (2017). Immunomodulating property of MAPK inhibitors: From translational knowledge to clinical implementation. Lab. Investig..

[99] Ascierto P.A., Dummer R. (2018). Immunological effects of BRAF+MEK inhibition. OncoImmunology.

[100] Kakavand H., Wilmott J.S., Menzies A.M., Vilain R., Haydu L.E., Yearley J.H., Thompson J.F., Kefford R.F., Hersey P., Long G.V. (2015). PD-L1 Expression and Tumor-Infiltrating Lymphocytes Define Different Subsets of MAPK Inhibitor-Treated Melanoma Patients. Clin. Cancer Res..

[101] Wilmott J.S., Haydu L.E., Menzies A.M., Lum T., Hyman J., Thompson J.F., Hersey P., Kefford R.F., Scolyer R.A., Long G.V. (2014). Dynamics of Chemokine, Cytokine, and Growth Factor Serum Levels in BRAF-Mutant Melanoma Patients during BRAF Inhibitor Treatment. J. Immunol..

[102] Ribas A., Lawrence D., Atkinson V., Agarwal S., Miller W.H., Carlino M.S., Fisher R., Long G.V., Hodi F.S., Tsoi J. (2019). Combined BRAF and MEK inhibition with PD-1 blockade immunotherapy in BRAF-mutant melanoma. Nat. Med..

[103] Dummer R., Lebbé C., Atkinson V., Mandalà M., Nathan P.D., Arance A., Richtig E., Yamazaki N., Robert C., Schadendorf D. (2020). Combined PD-1, BRAF and MEK inhibition in advanced BRAF-mutant melanoma: Safety run-in and biomarker cohorts of COMBI-i. Nat. Med..

[104] Ribas A., Algazi A., Ascierto P.A., Butler M.O., Chandra S., Gordon M., Hernandez-Aya L., Lawrence D., Lutzky J., Miller W.H. (2020). PD-L1 blockade in combination with inhibition of MAPK oncogenic signaling in patients with advanced melanoma. Nat. Commun..

[105] Gogas H., Dréno B., Larkin J., Demidov L., Stroyakovskiy D., Eroglu Z., Francesco Ferrucci P., Pigozzo J., Rutkowski P., Mackiewicz J. (2021). Cobimetinib plus atezolizumab in BRAF(V600) wild-type melanoma: Primary results from the randomized phase III IMspire170 study. Ann. Oncol..

[106] Varayathu H., Sarathy V., Thomas B.E., Mufti S.S., Naik R. (2021). Combination Strategies to Augment Immune Check Point Inhibitors Efficacy—Implications for Translational Research. Front. Oncol..

[107] Yeon M., Kim Y., Jung H.S., Jeoung D. (2020). Histone Deacetylase Inhibitors to Overcome Resistance to Targeted and Immuno Therapy in Metastatic Melanoma. Front. Cell Dev. Biol..

[108] Gibbons Johnson R.M., Dong H. (2017). Functional Expression of Programmed Death-Ligand 1 (B7-H1) by Immune Cells and Tumor Cells. Front. Immunol..

